# Fisetin Inhibits Human Melanoma Cell Invasion through Promotion of Mesenchymal to Epithelial Transition and by Targeting MAPK and NFκB Signaling Pathways

**DOI:** 10.1371/journal.pone.0086338

**Published:** 2014-01-23

**Authors:** Harish Chandra Pal, Samriti Sharma, Leah Ray Strickland, Santosh K. Katiyar, Mary E. Ballestas, Mohammad Athar, Craig A. Elmets, Farrukh Afaq

**Affiliations:** 1 Department of Dermatology, University of Alabama at Birmingham, Birmingham, Alabama, United States of America; 2 Comprehensive Cancer Center, University of Alabama at Birmingham, Birmingham, Alabama, United States of America; 3 Department of Pediatrics Infectious Disease, Children's of Alabama, School of Medicine, University of Alabama at Birmingham, Birmingham, Alabama, United States of America; Vanderbilt University, United States of America

## Abstract

Malignant melanoma is responsible for approximately 75% of skin cancer-related deaths. BRAF plays an important role in regulating the mitogen-activated protein kinase (MAPK) signaling cascade in melanoma with activating mutations in the serine/threonine kinase BRAF occurring in 60–70% of malignant melanomas. The BRAF-MEK-ERK (MAPK) pathway is a key regulator of melanoma cell invasion. In addition, activation of NFκB via the MAPK pathway is regulated through MEK-induced activation of IKK. These pathways are potential targets for prevention and treatment of melanoma. In this study, we investigated the effect of fisetin, a phytochemical present in fruits and vegetables, on melanoma cell invasion and epithelial-mesenchymal transition, and delineated the underlying molecular mechanism. Treatment of multiple human malignant melanoma cell lines with fisetin (5–20 µM) resulted in inhibition of cell invasion. BRAF mutated melanoma cells were more sensitive to fisetin treatment, and this was associated with a decrease in the phosphorylation of MEK1/2 and ERK1/2. In addition, fisetin inhibited the activation of IKK leading to a reduction in the activation of the NFκB signaling pathway. Treatment of cells with an inhibitor of MEK1/2 (PD98059) or of NFκB (caffeic acid phenethyl ester) also reduced melanoma cell invasion. Furthermore, treatment of fisetin promoted mesenchymal to epithelial transition in melanoma cells, which was associated with a decrease in mesenchymal markers (N-cadherin, vimentin, snail and fibronectin) and an increase in epithelial markers (E-cadherin and desmoglein). Employing three dimensional skin equivalents consisting of A375 cells admixed with normal human keratinocytes embedded onto a collagen-constricted fibroblast matrix, we found that treatment of fisetin reduced the invasive potential of melanoma cells into the dermis and increased the expression of E-cadherin with a concomitant decrease in vimentin. These results indicate that fisetin inhibits melanoma cell invasion through promotion of mesenchymal to epithelial transition and by targeting MAPK and NFκB signaling pathways.

## Introduction

Although melanoma represents the least common form of skin cancer (accounting for only about 5% of all skin cancer cases), it is the most deadly form of skin cancer claiming about 75% of skin cancer-related deaths [1]. Moreover, melanoma has a rapidly increasing incidence worldwide. According to an estimate from the American Cancer Society one person dies every hour from melanoma [2]. Furthermore, a total of 76,690 new cases of melanoma and 9,480 deaths have been projected to occur in the United States in 2013 [2]. Melanoma has a propensity to metastasize and patients with visceral metastasis have a median survival of six months.

Mutations that constitutively activate the serine/threonine kinase, BRAF (predominantely the oncogenic BRAF^V600E^) have been reported in 60–70% of malignant melanomas. In particular, BRAF^V600E^ mutations in melanoma are associated with increased invasion and metastasis of melanoma cells [3], [4]. In addition, oncogenic BRAF is also related to altered expression of extracellular matrix (ECM) genes and induction of epithelial-mesenchymal transition (EMT) [5]–[7]. Preclinical studies have demonstrated that BRAF plays an important role in regulating the mitogen-activated protein kinases (MAPK) signaling cascade by promoting proliferation, survival, and invasion of melanoma cells [8]–[14]. Once BRAF is activated, it further activates MEK1/2 MAP kinases that phosphorylate and translocate ERK1/2 [15]. ERK1/2 is constitutively activated in several cancer types including 90% of melanoma cases [16]. In addition to the BRAF-MEK-ERK (MAPK) pathway, the nuclear factor kappa B (NFκB) signaling pathway also plays an important role in cell invasion and is also found to be hyperactivated in variety of cancers including melanoma [17]–[20]. In melanoma, a potential mechanism by which NFκB signaling is constitutively activated is through the mutant BRAF pathway. Mutant BRAF activates the canonical pathway through activation of IKK which promotes phosphorylation and degradation of IκB resulting in translocation of NFκB into the nucleus [18]–[21]. In addition, MAPK also regulates NFκB signaling through MEK-induced activation of the IKK complex [22]. The role of the MAPK and NFκB pathways in melanoma cell survival, invasion and progression of EMT is being recognized. Thus these pathways are receiving attention as potential targets for the prevention and treatment of melanoma.

Fisetin is a naturally occurring flavonoid abundantly found in several fruits and vegetables, such as, strawberries, apples, persimmons, grapes, onions and cucumbers [23]. It possesses anti-proliferative [24], [25], pro-apoptotic [26]–[30], neuroprotective [31] and anti-oxidative activities [32]. Fisetin has been shown to inhibit MAPK and NFκB signaling pathways in different cancer cells [33]–[38]. In addition, the treatment of melanoma cells with fisetin induced MITF suppression by decreased expression of nuclear β-catenin with concomitant downregulation of the Wnt signaling pathway [39]. The goal of this study was to determine the effect of fisetin on melanoma cell invasion and to delineate the underlying molecular mechanism. Our results demonstrated that fisetin inhibits melanoma cell invasion by targeting the MAPK and NFκB signaling pathways in metastatic melanoma cells and in three-dimensional reconstituted human melanoma skin equivalents. Furthermore, fisetin inhibited melanoma cell invasion through promotion of mesenchymal to epithelial transition.

## Materials and Methods

### Materials

Fisetin (>98% pure), PD98059 and β-actin antibody were purchased from Sigma-Aldrich (St. Louis, MO). Caffeic acid phenethyl ester (CAPE) was purchased from MP Biologicals (Solon, OH). Three-dimensional skin equivalents of A375 melanoma cells (MLNM-FT-A375) were obtained from MatTek Corporation (Ashland, MA). The monoclonal and polyclonal antibodies for MEK1/2, phospho-MEK1/2 (Ser^217^/Ser^221^), ERK1/2 (phospho-p44/42, Thr^202^/Tyr^204^), E-cadherin, N-cadherin, vimentin and snail were obtained from Cell Signaling Technology (Beverly, MA). Monoclonal antibodies for NFκB p50, NFκB p65, IKKα, IκBα, phospho-IκBα, fibronectin and desmoglein were purchased from Santa Cruz Biotechnology, Inc. (Santa Cruz, CA). Anti-mouse, anti-goat and anti-rabbit secondary antibodies horseradish peroxidase conjugate were obtained from Millipore Corporation (Billerica, MA). Alexa Flour 488 or 594 labeled anti-mouse, anti-goat and anti-rabbit secondary antibodies were obtained from Life Technologies Corporation (Grand Island, NY).

### Cell Culture and Treatment

Human melanoma cells A375, RPMI-7951 and Hs294T were obtained from American Type Culture Collection (Manassas, VA). A375 and Hs294T cells were cultured as monolayers in DMEM medium (HyClone Laboratories Inc., Logan, UT) supplemented with 10% heat-inactivated fetal bovine serum and 100 mg/ml penicillin-streptomycin. RPMI-7951 cells were cultured in EMEM medium (Quality Biologicals Inc., Gaithersburg, MD) supplemented with 10% heat-inactivated fetal bovine serum and 100 mg/ml penicillin-streptomycin. SK-MEL-28 and SK-MEL-119 cells were obtained from Alan Houghton, Sloan-Kettering Institute for Cancer Research (New York, NY) and were cultured in RPMI1640 medium (HyClone Laboratories Inc., Logan, UT) supplemented with 10% heat-inactivated fetal bovine serum and 100 mg/ml penicillin-streptomycin [40]. The cells were maintained under standard cell culture conditions at 37°C and 5% CO_2_ in a humid environment. Fisetin (dissolved in DMSO) was used for the treatment of melanoma cells. The final concentration of DMSO used was 0.1% (v/v) for each treatment. For invasion assay cells were treated with fisetin (5–20 µM) or PD98059 (10–20 µM) or CAPE (10–20 µM) in serum-reduced medium. Melanoma cells were treated with fisetin in complete cell medium for western blot analysis.

### Cell Invasion Assay

To determine the anti-invasive potential of fisetin against BRAF mutated (A375, SK-MEL-28 and RPMI-7951), NRAS mutated (SK-MEL-119), and NRAS-BRAF wild type (Hs294T) melanoma cells Boyden chambers were used in which two chambers were separated by matrigel coated membranes. Melanoma cells (3×10^4^ cells/200 µl serum-reduced medium) were placed in the upper chamber of Boyden chamber containing 0, 5, 10 and 20 µM of fisetin. For additional experiments, A375 cells were treated with 10, 20 µM of fisetin, 10, 20 µM of MEK inhibitor (PD98059) or 10, 20 µM of NFκB (CAPE) for 24 hours. The lower chamber contained 110 µl of medium supplemented with 10% FBS. Chambers were assembled and kept in an incubator. Twenty four hours post treatment cells from the upper surface of membranes were removed with gentle swabbing and the invaded cells on the lower surface of membranes were fixed with chilled methanol and stained with crystal violet. Membranes were then washed and mounted onto glass slides and were examined microscopically.

### MEK Transfection

To examine whether the anti-invasive potential of fisetin is associated with MEK inhibition, MEK1 overexpression was induced in A375 cells by transfection. The day before transfection, A375 cells were trypsinized and counted. Approximately, 1.0×10^5^ cells in 1 ml complete growth medium were plated in 35 mm cell culture dish in order to achieve 50–70% confluent cells at the time of transfection. On the day of transfection, growth medium was replaced with 2 ml of complete growth medium. The MEK1-GFP plasmid was obtained from Addgene (Cambridge, MA) and expressed as described earlier [41]. The cells were transfected with 7.5 µg of MEK1-GFP or GFP-N2 control vector (Clontech Laboratories, Inc., Mountain View, CA) mixed with Xfect Reaction Buffer (Clontech Laboratories, Inc.) to a final volume of 100 µl and 2.1 µl Xfect Polymer. Forty-eight hours after transfection, the cells were harvested for invasion assay.

### Treatment of Tissue-engineered Three-dimensional Skin Equivalents

The human full thickness melanoma (MLNM-FT-A375) tissues were generated by seeding human metastatic melanoma A375 cells and normal human epidermal keratinocytes at a ratio of 1∶5 in a cell culture insert purchased from MatTek (Ashland, MA). These were cultured to form highly differentiated three-dimensional skin equivalents in MLNM-FT-MM medium at 37°C and 5% CO_2_ in a humidified chamber [42], [43]. At day seven, three-dimensional skin equivalents containing A375 cells were treated with fisetin (5–20 µM) for 7 days. Tissue samples were treated with fisetin in 0.05% DMSO in MLNM-FT-MM medium. The media was replaced every alternate day. Throughout the experiment the three-dimensional skin equivalents were maintained in six well culture plates at air-liquid interface with the lower dermal side of the tissue exposed to media and the upper epidermal stratum corneum exposed to air. To determine the mechanism of inhibition of melanoma cell invasion, tissue-engineered three-dimensional skin equivalents of A375 cells were treated with 20 µM fisetin or 20 µM MEK inhibitor (PD98059) or 20 µM NFκB inhibitor (CAPE) for 12 days. Tissue samples were treated with fisetin, PD98059 or CAPE in 0.05% DMSO in MLNM-FT-MM medium. After treatment skin samples were collected, fixed in 10% neutralized formalin and embedded in paraffin. For histochemsitry 5 µm sections were cut, deparaffinized in xylol and rehydrated through graded ethanol then H&E was performed. The skin sections were examined microscopically.

### Preparation of Total Cell Lysate

The melanoma cells (A375 and RPMI-7951) were treated with fisetin (5–20 µM; 24 hours). After the 24 hours incubation period, the medium was aspirated and the cells were washed with PBS (10 mmol/l, pH 7.45). The cells were then incubated in an ice cold lysis buffer containing 10 mM HEPES (pH 7.9), 100 mM KCl, 10 mM EDTA, 20 mM EGTA, 100 mM DTT, 20 mM PMSF, 0.5% NP-40 with freshly added protease inhibitors (leupeptin, aprotinin and benzamidine) for 20 min. The cells were then scraped and the lysate was collected in a microfuge tube and passed through a 21.5-G needle to break up the cell aggregates. The lysate was cleared by centrifugation at 14,000 g for 10 min at 4°C, and the supernatant (total cell lysate) collected, aliquoted and was used on the day of preparation or immediately stored at −80°C for use at a later time.

### Preparation of Cytosolic and Nuclear Lysate

Following treatment of A375 and RPMI-7951cells with fisetin (5–20 µM; 24hours), the medium was aspirated and the cells were washed twice in PBS (10 mM, pH 7.4). The cells were incubated in 250 µl ice-cold lysis buffer containing 10 mM HEPES (pH 7.9), 10 mM KCl, 0.1 mM EDTA, 0.1 mM EGTA, 1 mM DTT, 1 mM PMSF, 0.5% NP-40 with freshly added protease inhibitors (leupeptin, aprotinin and benzamidine) for 20 min. The cells were then scraped and the lysate was collected in a microfuge tube, mixed on a vortex and then centrifuged for 1 min (14,000 g) at 4°C. The supernatant was saved as cytosolic lysate and stored at −80°C. The nuclear pellet was resuspended in 50 µl of ice-cold nuclear extraction buffer containing 20 mM HEPES (pH 7.9), 0.4 M NaCl, 1 mM EDTA, 1 mM EGTA, 1 mM DTT, 1 mM PMSF, 0.5% NP-40 with freshly added protease inhibitors (leupeptin, aprotinin and benzamidine) for 30 min with intermittent mixing. The tubes were centrifuged for 5 min (14,000 g) at 4°C, and the supernatant (nuclear extract) was stored at −80°C for use at a later time.

### Western Blot Analysis

For western blotting, 30–50 µg protein was resolved over 8–12% Tris–glycine gels and transferred onto a polyvinylidene fluoride (PVDF) membrane. The non-specific sites on blots were blocked by incubating in blocking buffer (5% non-fat dry milk/0.1% Tween 20 in Tris-Buffered Saline (TBS), pH 7.6) for 1 hour at room temperature, then incubated with appropriate primary antibody in blocking buffer for overnight at 4°C, followed by 1 hour incubation at room temperature with anti-mouse or anti-rabbit secondary antibody horse-radish peroxidase conjugate. Membranes were then washed with TBST washing buffer and detected by chemiluminescence using Pierce ECL Western Blotting Substrate (Thermo Scientific, Rockford, IL) and autoradiography using HyBlot CL Autoradiography Film obtained from Densville Scientific Inc., (Metuchen, NJ). Densitometric measurements of the band in Western blot analysis were performed using ImajeJ scientific software program (http://rsbweb.nih.gov/ij/index.html).

### Immunofluorescence Staining

After treatment with 10 and 20 µM fisetin for 12 days, tissue from three-dimensional skin equivalents were collected, fixed in 10% neutralized formalin and embedded in paraffin. For immunofluorescence staining, 5 µm sections were cut deparaffinized and rehydrated and were heated at 95°C for 30 min in citrate buffer (pH 6.0) for antigen retrieval. After blocking for 30 min with blocking buffer sections were incubated with primary antibody against E-cadherin or vimentin overnight at 4°C followed by incubation with specific Alexa Flour 488 or 594 labeled secondary antibodies for 1 hour at room temperature in the dark. After washing, the sections were incubated with vectashield mounting media containing DAPI for 10 min in the dark and analyzed under microscope immediately.

### Statistical Analysis

The results are expressed as the mean ± SEM. Statistical analysis was performed by Student’s t-test. The *P* value <0.05 was considered statistically significant.

## Results

### Fisetin Treatment Reduces Invasion of Melanoma Cells

**Figure 1:**
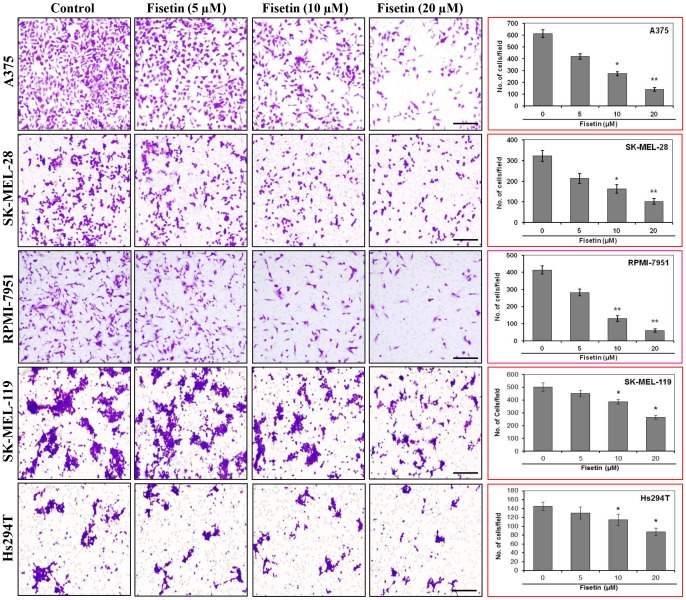
Effect of fisetin treatment on invasion of melanoma cells. The invasive capacity of BRAF mutated (A375, SK-MEL-28 and RPMI-7951), NRAS mutated (SK-MEL-119) and BRAF-NRAS wild type (Hs294T) melanoma cells was determined *in vitro* using Boyden chamber assay. Melanoma cells (3×10^4^ cells/200 µl serum-reduced medium) were placed in the upper chamber of Boyden chamber containing 0, 5, 10 and 20 µM of fisetin. The lower chamber contained 110 µl of medium supplemented with 10% FBS. After 24 hours of incubation, the invaded cells on the lower surface of the membranes were fixed with chilled methanol and stained with crystal violet. A representative picture from three independent experiments is shown. The invaded cells were counted in at least four to five randomly selected microscopic fields on the membrane and the results are summarized and expressed as the mean number of invaded cells ± SEM per microscopic field. Significant difference *versus* control group, *P<0.05, **P<0.01. Bar = 100 µm.

Since cell invasion is a key step involved in tumor metastasis [44], inhibition of cell invasion by fisetin may represent an important strategy in the prevention/treatment of melanoma. By employing Boyden chamber cell invasion assay, we assessed whether treatment of BRAF mutated melanoma cell lines (such as A375, SK-MEL-28 and RPMI-7951) with fisetin inhibits their invasive potential. For these studies, we selected concentrations of fisetin (5–20 µM) that do not have significant effect on growth of melanoma cells (data not shown). Fisetin at higher doses (>20 µM) inhibits growth of metastatic melanoma cells with minimal cytotoxic effects on normal human melanocytes and normal human epidermal keratinocytes (data not shown). As shown in Figure 1, treatment of cells with fisetin (5–20 µM) for 24 hours reduced the invasive potential of A375, SK-MEL-28 and RPMI-7951 melanoma cells in a dose-dependent manner compared to their respective untreated control cells. The density of invaded cells on the membrane and the number of invaded cells/microscopic field are shown in Figure 1. The cell invasion was inhibited by 30 to 77% (P<0.05–0.01), 34 to 68% (P<0.05–0.01), and 32 to 85% (P<0.01) in A375, SK-MEL-28 and RPMI-7951 cells respectively (Figure 1). In addition, we also utilized NRAS mutant SK-MEL-119 cells and NRAS-BRAF wild type Hs294T cells to determine the effect of fisetin on cell invasion. Cell invasion was inhibited by 10 to 47% (P<0.05) in SK-MEL-119, and by 10 to 39% (P<0.05) in Hs294T cells (Figure 1). These data suggest that BRAF mutant cells were more sensitive to fisetin treatment than NRAS mutant cells or NRAS-BRAF wild type cells.

### Fisetin Treatment Inhibits Invasion of Melanoma Cells in Three-dimensional Human Skin Equivalents

**Figure 2:**
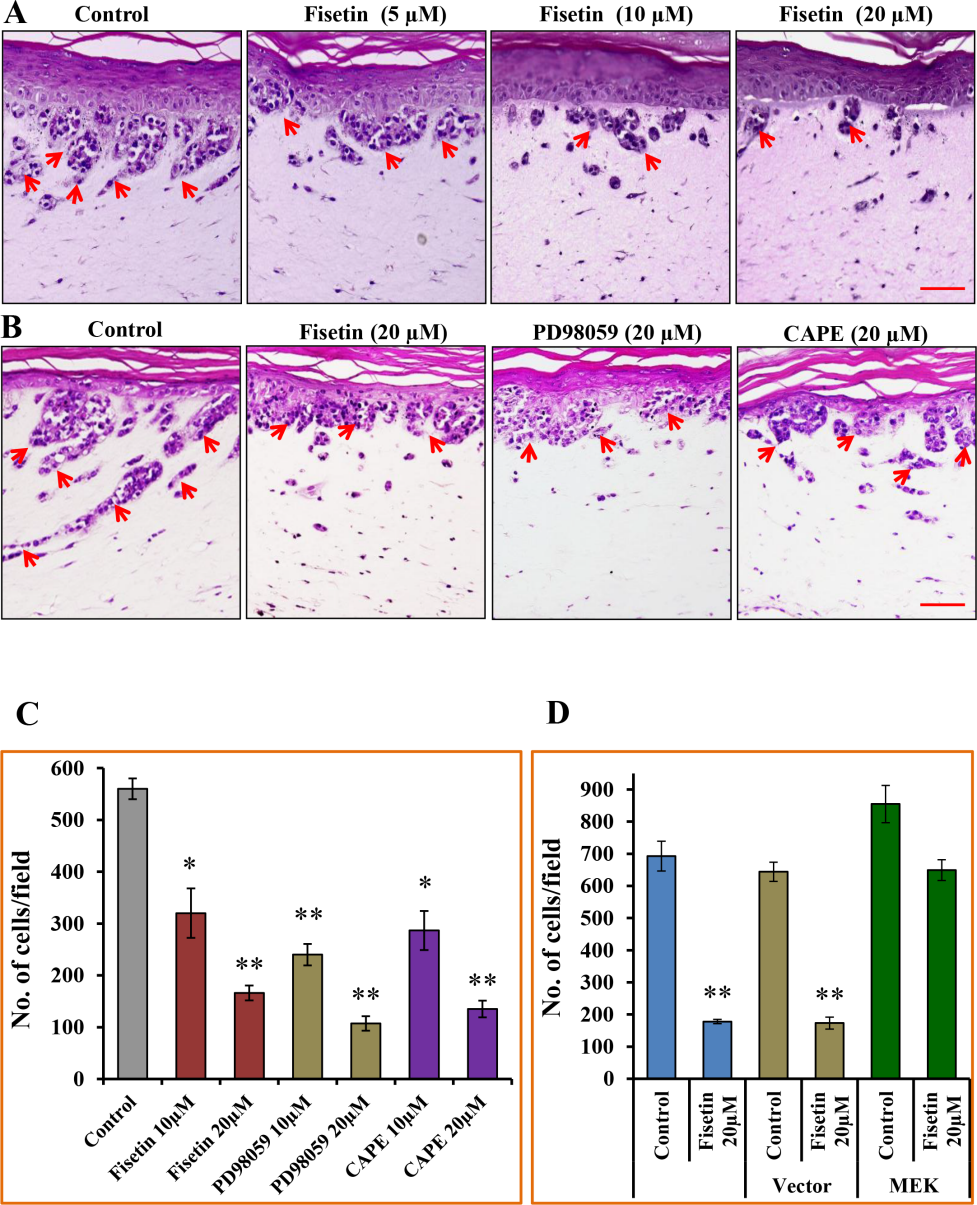
Effect of Fisetin, MEK and NFκB inhibitor on invasion of A375 cells. [**A**] The three-dimensional skin equivalents containing A375 cells were treated with fisetin (5–20 µM) for 7 days. After treatment with fisetin, skin samples were collected and H&E was performed. A representative picture from three independent experiments is shown. Arrows indicate invading A375 cells. Bar = 25 µm. [**B**] The three-dimensional skin equivalents containing A375 cells were treated with 20 µM of fisetin, PD98059 or CAPE for 12 days. After treatment samples were collected and H&E was performed. A representative picture from three independent experiments is shown. Arrows indicate invading A375 cells. Bar = 25 µm. [**C**] A375 cells (3×10^4^ cells/200 µl serum-reduced medium) were placed in the upper chamber of Boyden chamber containing 10 and 20 µM of fisetin, PD98059 or CAPE. The lower chamber contained 110 µl of medium supplemented with 10% FBS. After 24 hours of incubation, the invaded cells on the lower surface of the membranes were fixed with chilled methanol and stained with crystal violet. The invaded cells were counted on the membrane in at least four to five randomly selected microscopic fields. The results are summarized and expressed as the mean number of invaded cells ± SEM per microscopic field. Significant difference *versus* control group, *P<0.05, **P<0.01. [D] A375 cells were transfected with MEK1-GFP or GFP-N2 control vector using Xfect Transfection Reagent as per the manufacturer’s protocol. Forty-eight hours after transfection, the cells were harvested and invasion assay was performed as described in “Materials and Methods” section. Significant difference *versus* control group, **P<0.01.

To establish the relevance of the anti-invasive potential of fisetin, we used three-dimensional human melanoma skin equivalents comprised of A375 cells admixed with normal human keratinocytes embedded onto a collagen-constricted fibroblast matrix. In control samples that were not treated with fisetin a progressive development of nodes at the epidermal-dermal junction and vertical invasion of metastatic melanoma A375 cells to the dermis was observed (Figure 2A). However, treatment with fisetin (5–20 µM) reduced the size of nodes as well as invasive characteristic of A375 cells in a dose-dependent manner (Figure 2A).

### Fisetin Treatment Inhibits Melanoma Cell Invasion by Targeting MEK1/2 and NFκB

To determine whether fisetin abrogates melanoma cell invasion by inhibiting MEK1/2 and NFκB, we compared the effect of inhibitor of MEK1/2 (PD98059) and NFκB (CAPE) with fistein. BRAF mutated A375 cells were treated with PD98059 (10–20 µM) or CAPE (10–20 µM) or fisetin (10–20 µM) for 24 hours. The doses of MEK1/2 and NFκB inhibitors used in this study had no significant effect on the growth of A375 cells (data not shown). As shown in Figure 2C, we found that MEK1/2 and NFκB inhibitors reduced cell invasion by 57 to 81% (P<0.01), and 49 to 76% (P<0.05–0.01) respectively, when compared to fisetin (43–76%; P<0.05–0.01). These results were further confirmed by treating three-dimensional human melanoma skin equivalents comprised of A375 cells with 20 µM of fisetin, MEK1/2 and NFκB (CAPE) inhibitors for 12 days. Treatment with fisetin or a MEK1/2 or NFκB inhibitor decreased the progression and development of nodes as well as a reduction in invasive characteristics of A375 cells in skin equivalents when compared with control (Figure 2B). These results suggest that fisetin could reduce cell invasion, at least in part by inhibition of MEK1/2 and NFκB. To test whether constitutive activation of MEK rescues the inhibition of melanoma invasion by fisetin, for this purpose MEK overexpression was induced in A375 cells. We found that MEK overexpression resulted in increased invasion of A375 cells. Fisetin treatment did not significantly reduce invasion of MEK overexpressing A375 cells. However, fisetin treatment did result in significant reduction in invasion of vector transfected A375 cells when compared to control (Figure 2D).

### Fisetin Treatment Inhibits Phoshorylation of MEK1/2 and ERK1/2 in Melanoma Cells

**Figure 3:**
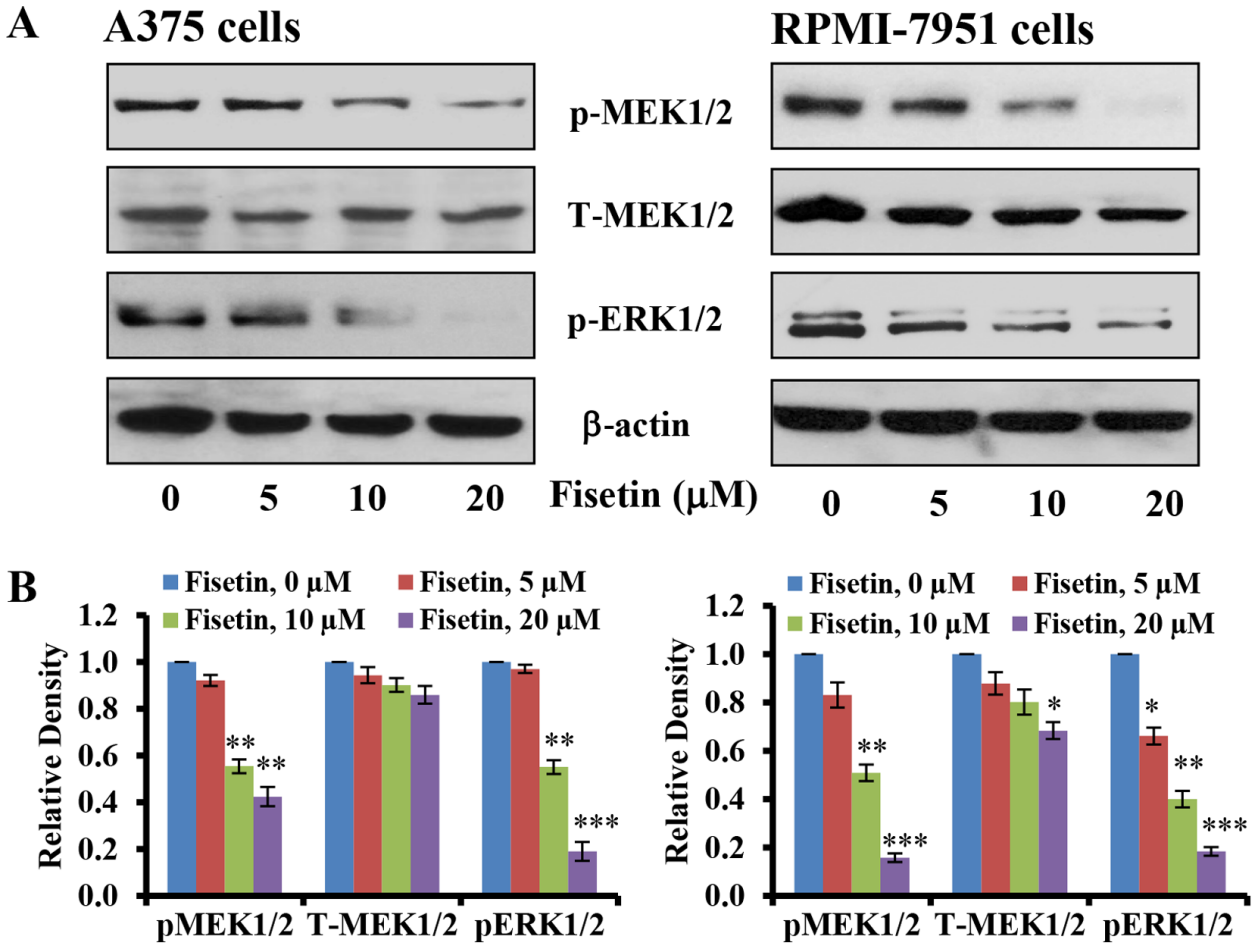
Effect of fisetin treatment on phoshorylation of MEK1/2 and ERK1/2 in melanoma cells. The melanoma cells (A375 and RPMI-7951) were treated with fisetin (5–20 µM; 24 hours) and then the cells were harvested. Total cell lysates were prepared and [**A**] Western blot analysis for protein expression and [**B**] relative density was performed, as described in the ‘Materials and Methods’ section. Equal loading of protein was confirmed by stripping the immunoblot and reprobing it for β-actin. The immunoblots shown here are representative of three independent experiments with similar results, shown in relative units ± SEM. Significant difference *versus* control group, *P<0.05, **P<0.01 and ***P<0.001.

Activated BRAF in the MAPK signal transduction pathway propagates signaling by phosphorylating MAPKKs, MEK1/2 [16], [22]. The downstream substrates of MEK1/2 are ERK1/2, which regulate an array of proteins involved in cell invasion [15], [16], [22]. Therefore, we next investigated whether the reduction of cell invasion by fisetin was due to inhibition in phosphorylation of MEK1/2 and ERK1/2. Our data indicate that treatment of A375 and RPMI-7951 cells with fisetin (5–20 µM; 24 hours) resulted in a significant dose-dependent-decrease in the phosphorylation of MEK1/2 (Figure 3A–B). In addition, fisetin treatment significantly reduced the phosphorylation of ERK1/2 in a dose-dependent manner as shown by western blot and relative density of the bands (Figure 3A–B).

### Fisetin Treatment Inhibits Nuclear Translocation of NFκB

**Figure 4:**
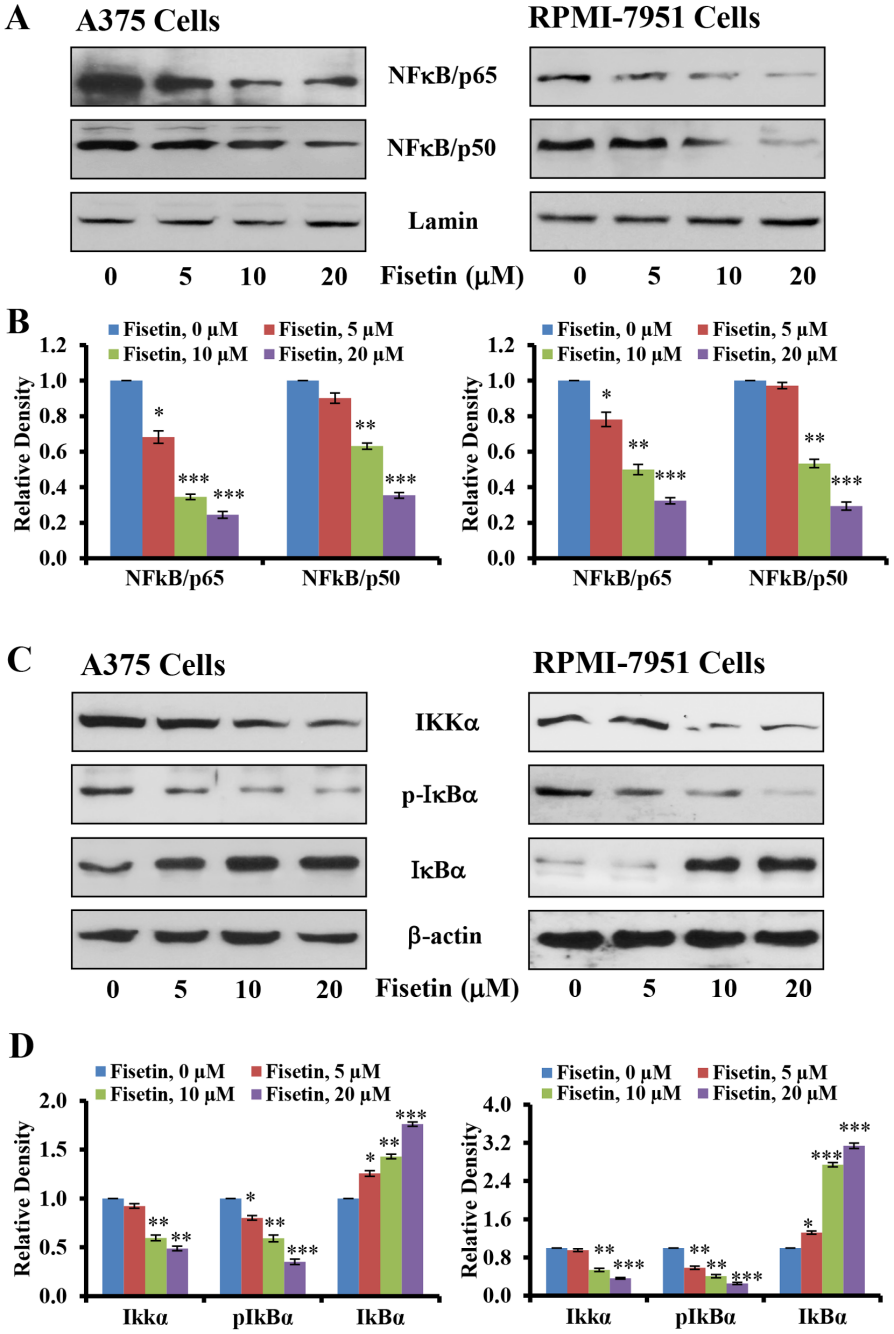
Effect of fisetin treatment on nuclear translocation of NFκB subunits, activation of IKKα and phosphorylation and degradation of IκBα in melanoma cells. The melanoma cells (A375 and RPMI-7951) were treated with fisetin (5–20 µM; 24 hours) and then the cells were harvested. Nuclear and cytosolic lysates were prepared and [A&C] Western blot analysis for protein expression and [B&D] relative density was performed, as described in the ‘Materials and Methods’ section. Equal loading of protein was confirmed by stripping the immunoblot and reprobing it for Lamin (nuclear fraction) and β-actin (cytosolic fraction). The immunoblots shown here are representative of three independent experiments with similar results, shown in relative units ± SEM. Significant difference *versus* control group, *P<0.05, **P<0.01 and ***P<0.001.

Since activation of NFκB via the MAPK pathway is regulated through MEK-induced activation of IKK, we next determined whether fisetin treatment could inhibit constitutive activation of the NFκB signaling pathway [12], [17]–[20]. As shown in Figure 4A–B, our data clearly demonstrated that fisetin (5–20 µM; 24 hours) treatment of A375 and RPMI-7951 cells significantly reduced nuclear translocation of NFκB p65 and NFκB p50 subunits. Studies have shown that the activation and nuclear translocation of NFκB is because of its dissociation from the inhibitory protein IκBα [18], [21], [45]. Therefore, we next examined the effect of fisetin on the phosphorylation and degradation status of IκBα in A375 and RPMI-7951 cells. We found that fisetin treatment significantly reduced the phosphorylation and degradation of IκBα in a dose-dependent manner as shown by western blot and relative density of the bands (Figure 4C–D). Studies have demonstrated that IKK phosphorylates IκBα and plays an important role in its degradation. We therefore measured the levels of IKKα protein and found that fisetin treatment significantly reduced the protein expression of IKKα in both these melanoma cell lines in a dose-dependent manner (Figure 4C–D).

### Fisetin Treatment Inhibits EMT in Melanoma Cells

**Figure 5:**
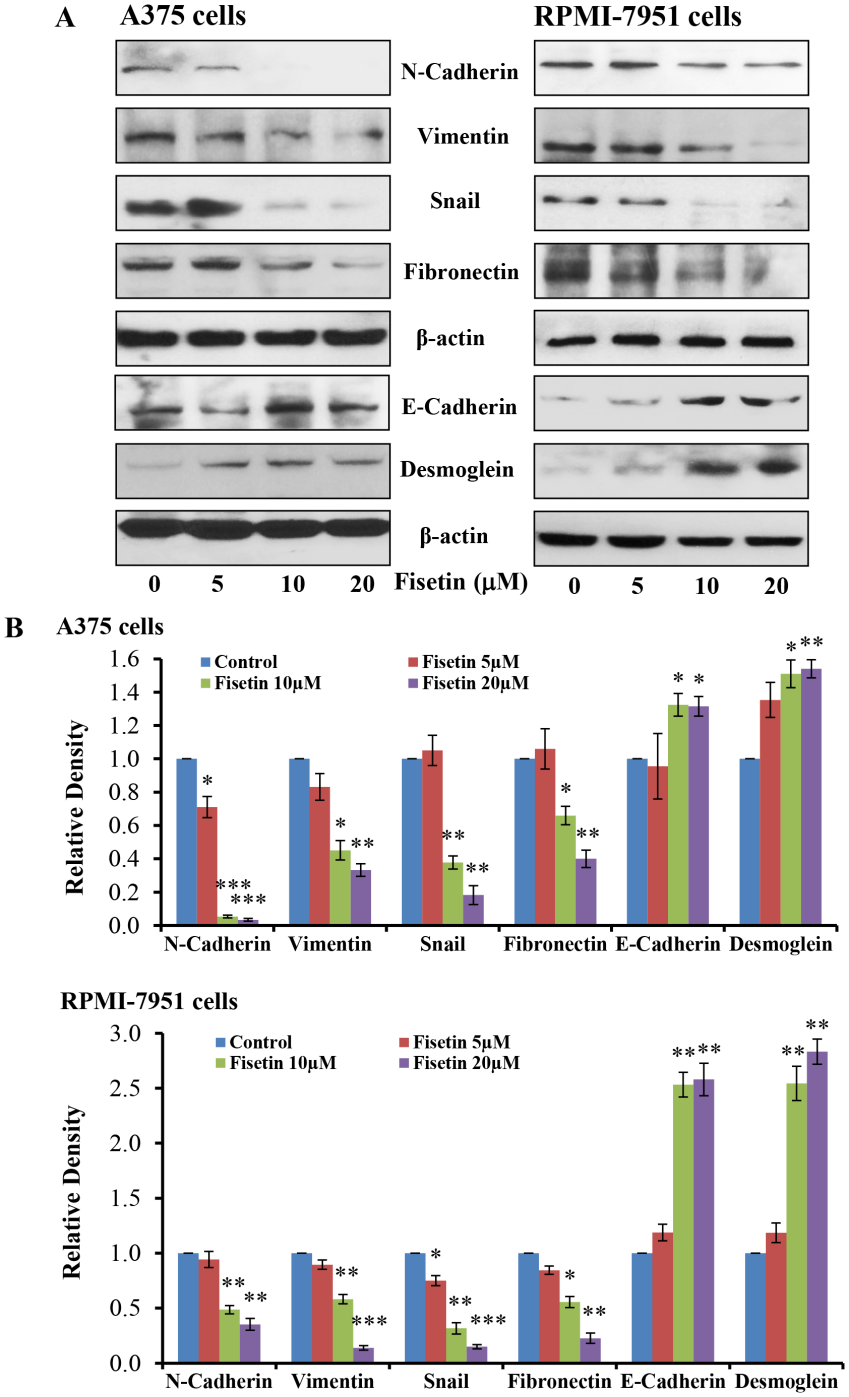
Effect of fisetin treatment on epithelial and mesenchymal marker proteins expression in melanoma cells. The melanoma cells (A375 and RPMI-7951) were treated with fisetin (5–20 µM; 24hours) and then cells were harvested. Total cell lysates were prepared and [**A**] Western blot analysis for protein expression and [**B**] relative density was performed, as described in the ‘Materials and Methods’ section. Equal loading of protein was confirmed by stripping the immunoblot and reprobing it for β-actin. The immunoblots shown here are representative of three independent experiments with similar results, shown in relative units ± SEM. Significant difference *versus* control group, *P<0.05, **P<0.01 and ***P<0.001.

In malignant melanoma activation of the BRAF-MEK-ERK and NFκB signaling pathways leads to induction of epithelial to mesenchymal transition (EMT) resulting in cell invasion [12], [17]–[20]. During EMT, the polarized epithelial cells acquire certain attributes of mesenchymal cells, and thus are able to penetrate the mesenchymal layer through the basement membrane and invade neighboring tissues [44]. Acquisition of EMT is associated with epithelial integrity, invasion and ultimately metastasis; therefore we determined the effect of fisetin (5–20 µM; 24 hours) treatment on mesenchymal and epithelial marker proteins in A375 and RPMI-7951 cells. Fisetin treatment significantly reduced mesenchymal marker proteins such as N-cadherin, vimentin, snail and fibronectin in these melanoma cell lines (Figure 5A–B). The transcription factor snail is implicated in transcriptional repression of E-cadherin, we therefore studied the effect of fisetin on E-cadherin protein expression, and found that fisetin treatment significantly increased the protein expression of E-cadherin (Figure 5A–B). In addition, fisetin treatment significantly increased the protein expression of desmoglein in both these melanoma cell lines as shown by western blot analysis (Figure 5A–B).

### Fisetin Treatment Increases E-cadherin and Decreases Vimentin Protein Expression in Tissue-engineered Three-dimensional Human Skin Equivalents

**Figure 6:**
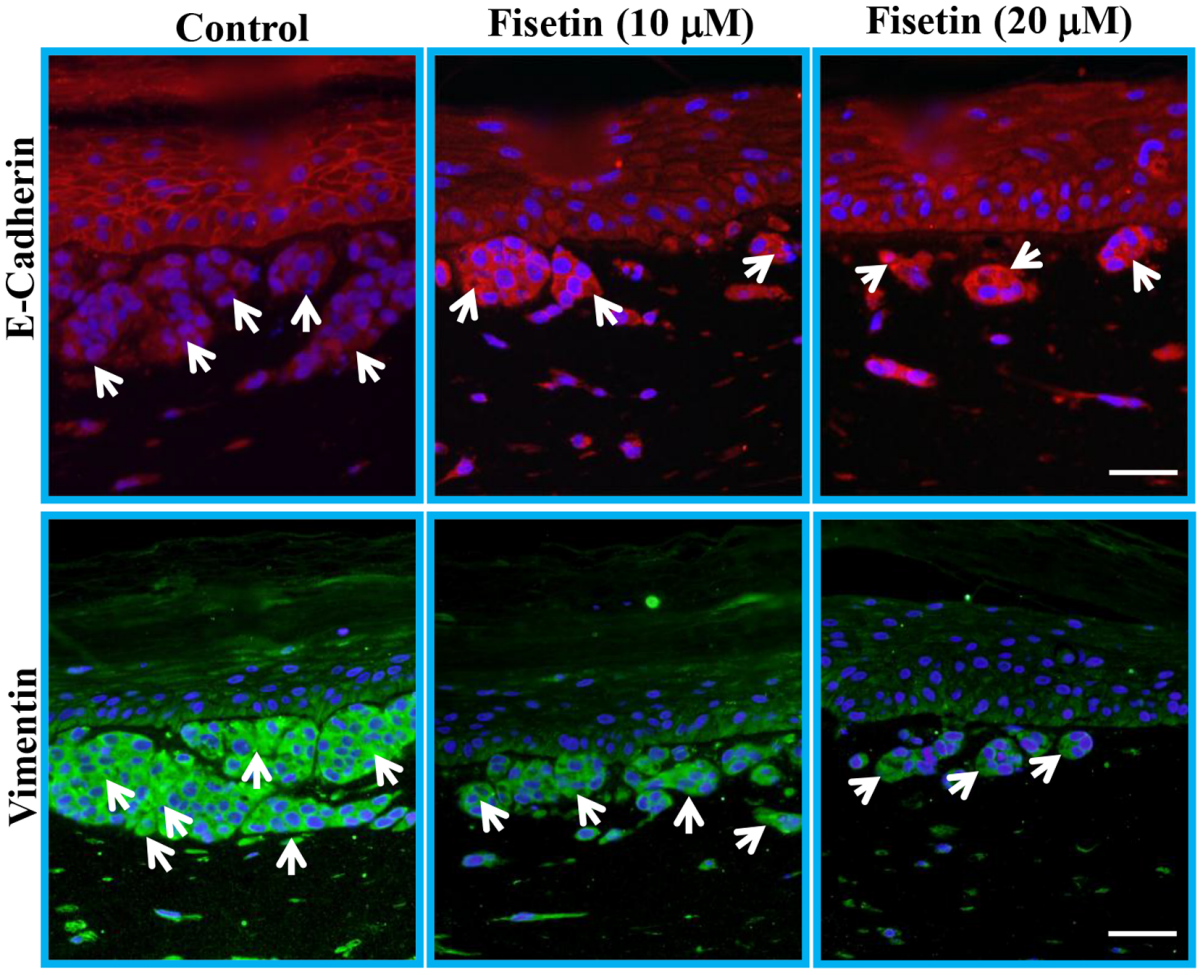
Effect of fisetin treatment on E-cadherin and vimentin protein expression in tissue-engineered three-dimensional skin equivalents. The tissue-engineered three-dimensional skin equivalents consisting A375 cells were treated with (10 and 20 µM fisetin for 7 days. After treatment, samples were collected, fixed in formalin and paraffin blocks were prepared. Five micormeter sections were cut, deparaffinized, rehydrated and were heated at 95°C for 20 min in citrate buffer (pH 6.0) for antigen retrieval. Sections were incubated with primary antibody against E-cadherin or vimentin overnight at 4°C followed by incubation with specific Alexa Flour 488 or 594 labeled secondary antibody for 1 hour at room temperature in the dark. After washing, the sections were incubated with vectashield mounting media containing DAPI for 10 min in the dark and analyzed under microscope immediately. A representative picture from three independent experiments is shown. E-cadherin is shown in red, vimentin in green and DAPI in blue. Bar = 25 µm.

We also used three-dimensional skin equivalents consisting of A375 cells admixed with normal human keratinocytes embedded onto a collagen-constricted fibroblast matrix to examine the effect of fisetin. E-cadherin and vimentin expression are respective hallmarks of epithelial and mesenchymal characteristics. In untreated three-dimensional skin equivalents there was weak expression of E-cadherin in the invading melanoma cells as shown by arrow (Figure 6). However, fisetin (10–20 µM; 7days) treatment increased the protein expression of E-cadherin in A375 melanoma cells and very few cells were observed in the nodes (Figure 6). In addition, fisetin treatment inhibited the protein expression of mesenchymal protein vimentin in invading A375 cells when compared with highly invaded nodes of control (Figure 6). These results suggest that fisetin reduced the invasive potential of A375 by increasing the protein expression of E-cadherin and decreasing the protein expression of vimentin.

## Discussion

The metastatic ability of melanoma is related to a poor prognosis and shorter survival. During the process of invasion melanoma cells lose cell-cell adhesion, acquire mobility, and leave the site of the primary tumor to invade adjacent tissues and form metastases. Recent studies suggest that the BRAF^V600E^ mutation in melanoma is associated with increased migratory and metastatic capacity [3], [4]. Furthermore, a growing body of evidence supports that BRAF-MEK-ERK (MAPK) and NFκB pathways play an important role in invasion and metastasis since they are constitutively activated in melanoma [8]–[14]. Melanoma is refractory to current chemotherapeutic treatment; in addition these agents also come with a high burden of toxicities. Therefore, the most promising strategy to prevent melanoma cell invasion and metastasis is by targeted inhibition of these signaling processes through non-toxic dietary agents that have anti-invasive potential.

In the present study, we found that fisetin, a non-toxic dietary flavonoid, greatly reduced the invasive potential of melanoma cells (Figure 1). More importantly, fisetin inhibited the invasion of melanoma cells and formation of nodes in three-dimensionally reconstituted human melanoma skin equivalents (Figure 2). This represents an environment closer to *in vivo* conditions where melanoma cells gain mobility and leave the site of the primary tumor to invade adjacent tissues. BRAF mutant melanoma cells were more sensitive to fisetin treatment and this was associated with inhibition of MEK1/2 phosphorylation (Figure 3). More importantly inhibition of MEK1/2 phosphorylation by fisetin resulted in reduced phosphorylation of ERK1/2 (Figure 3). ERK1/2 is constitutively activated in melanomas [16] and correlates with high frequencies of activating BRAF mutations. The NFκB signaling pathway is also found to be hyper-activated in melanoma and plays an important role in cell survival, invasion and metastasis [12], [17]–[20]. In melanoma mutant BRAF activates the NFκB signaling pathway [18]–[21]. In addition, MAPK also regulates NFκB signaling through MEK-induced activation of the IKK complex [22]. Thus inhibition of MAPK and NFκB could abrogate melanoma invasion and metastasis. In the present study, fisetin greatly reduced the translocation of NFκB p65 and p50 in to the nucleus (Figure 4) suggesting the potential mechanism of its anti-invasive activity. NFκB signaling is activated through IKK (IκB kinase) which activates NFκB signaling by initiating the phosphorylation and degradation of IκBα by the proteasome resulting in translocation of NFκB to the nucleus [18], [21], [45]. In the nucleus NFκB promotes metastasis through upregulation of metastasis inducer genes [19], [46]. We found fisetin inhibited the protein expression of IKKα, which in turn reduced the phosphorylation of IκBα (Figure 4). Inhibition of IκBα phosphorylation by fisetin prevented further degradation of IκBα by the proteasome as observed by an increased expression of IκBα in fisetin treated melanoma cells (Figure 4). These results clearly demonstrated that the anti-invasive potential of fisetin against BRAF mutated cells is directly related to inhibition of MAPK and NFκB signaling.

Tumor progression, invasion and metastasis occur when epithelial cells are converted to highly migratory and invasive mesenchymal-like cells in a highly regulated multistep process known as EMT [44]. In essence, during EMT cells lose their epithelial characteristics and acquire a mesenchymal phenotype. This transition is characterized by a loss of expression of epithelial biomarkers, such as E-cadherin and cytokeratins with an accompanying increase in expression of mesenchymal biomarkers such as N-cadherin and vimentin [47]–[50]. Functional loss of E-cadherin is associated with metastatic dissemination, invasion and a poor prognosis; it is thus considered one of the hallmarks of EMT [51]. Loss of E-cadherin expression is common in melanoma and has a critical role in altering melanoma cell interactions and promoting tumor cell invasion and metastasis [52]–[54]. We found that fisetin treatment increased the expression of E-cadherin protein in melanoma cells as well as in three-dimensionally reconstituted human melanoma skin equivalents (Figures 5 and 6). Fisetin treatment also reduced the protein expression of desmoglein (Figure 5), a member of the cadherin family which is involved in the formation of desmosomes, structures that join one cell to another cell to maintain epithelial membrane integrity. Thus fisetin has the potential to inhibit the process of EMT. It has been shown that during EMT the inhibition of E-cadherin expression leads to induction of N-cadherin expression and has been associated with tumor invasiveness [55]–[57]. In the present study, fisetin reduced the expression of N-cadherin in melanoma cells (Figure 5). Another important mesenchymal biomarker is vimentin, an intermediate filament normally expressed in mesenchymal cells that is involved in the migration of epithelial cells during development [58]. The expression of vimentin in cancer cells is believed to enhance migration and invasiveness [59]. Expression of vimentin is characteristic of epithelial cells undergoing the EMT process and is related to a reduced expression of E-cadherin and upregulation of N-cadherin [57]–[61]. In addition, increased expression of vimentin in various cancers is correlated with a poor prognosis [57]. In the present study, fisetin reduced the expression of vimentin in melanoma cells and as well as in three-dimensionally reconstituted human melanoma skin equivalents (Figures 5 and 6). Furthermore, snail, a member of the superfamily of transcriptional repressor of E-cadherin expression, has been reported to play important role in promotion of cell motility, invasiveness and progression of EMT in several cancers including melanoma [62]–[66]. We found that fisetin decreased the expression of snail in both melanoma cell lines (Figure 5). Regulation of EMT is integrated in the upstream activation of snail transcription by NFκBp65 [36], [67], [68]. Thus snail is also required for the metastatic potential of melanoma and acts through induction and maintenance of EMT [13], [14], [68]. Therefore, inhibition of snail expression in the present study is a consequence fisetin-induced inhibition of NFκB (Figure 4). In addition, fisetin also inhibited the protein expression of fibronectin (Figure 5), an extra cellular matrix protein involved in cell adhesion and migration in BRAF mutated melanomas [6]–[8]. These results clearly demonstrated that fisetin inhibited progression of EMT by inhibiting mesenchymal markers and inducing epithelial markers.

**Figure 7:**
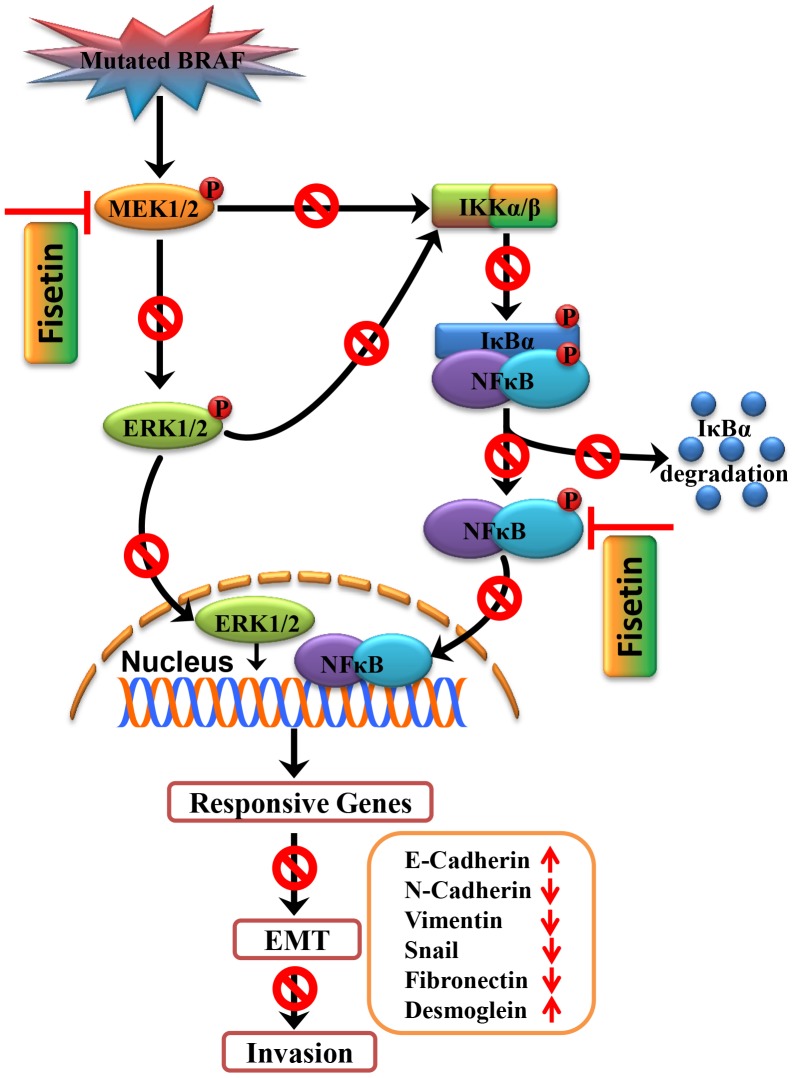
Proposed mechanism of fisetin for inhibition of melanoma cell invasion through promotion of mesenchymal to epithelial transition and by targeting MAPK and NFκB signaling pathways.

Our study identified fisetin, a flavonoid abundantly present in fruits and vegetables, as an effective inhibitor of melanoma cell invasion. The anti-invasive activity of fisetin occurred through inhibition of BRAF-MEK-ERK (MAPK) and NFκB signaling pathways (Figure 7), which are aberrantly activated in a majority of metastatic melanomas. In addition, fisetin promoted mesenchymal to epithelial transition leading to reduction in cell invasion. Further mechanism-based *in vivo* studies are needed to establish the use of fisetin either alone or in combination with anti-metastatic drugs for the management of melanoma cell invasion and metastasis.
