# Expression of Concern: Fisetin Inhibits Human Melanoma Cell Invasion through Promotion of Mesenchymal to Epithelial Transition and by Targeting MAPK and NFκB Signaling Pathways

**DOI:** 10.1371/journal.pone.0313108

**Published:** 2024-10-28

**Authors:** The PLOS ONE Editors

Following the publication of this article [1], concerns were raised regarding results presented in Fig 1. Specifically, the following panels appear to partially overlap, despite being used to represent different experimental conditions:

- SK-MEL-28 Control and SK-MEL-28 Fisetin (5 µM)
- RPMI-7951 Control and RPMI-7951 Fisetin (5 µM)

The corresponding author did not provide an explanation for the partial image overlap, but they did provide a revised Fig 1 in which the SK-MEL-28 Fisetin (5 µM) and RPMI-7951 Fisetin (5 µM) panels are replaced (provided in S1 File) and the individual level data underlying the Fig 1 graphs for the A375, SK-MEL-28, and RPMI-7951 (provided in S2 File). The authors stated that the individual level data for the SK-MEL-119 and Hs294T results are no longer available. The raw image data underlying the Fig 1 results were not provided for editorial review. In the absence of the complete underlying data, the concerns with Fig 1 cannot be fully resolved.

In addition to the above concerns, it was also noted that panels presented in Figs 3, 4, and 5 of this article [1] appear similar to results presented in articles [2 and 3, retracted in 4] published several years after this article [1]; the corresponding author of [1] stated that they did not collaborate or work together with the authors of [2 and 3, retracted in 4], and provided the available cropped underlying blot data for the results presented in Figs 3, 4, and 5 of [1], which are provided in the S3 File available with this notice. PLOS considers the concerns with Figs 3, 4, and 5 in this article [1] resolved.

In light of the concerns raised with Fig 1, which cannot be fully resolved in the absence of the complete underlying data, the *PLOS ONE* Editors issue this Expression of Concern to notify readers of the concerns and to relay the available data provided by the corresponding author.

## Supporting information

S1 File Fig 1 with replacement panels for the SK-MEL-28 Fisetin (5 µM) and RPMI-7951 Fisetin (5 µM) results (PDF)

S2 File Individual level data underlying the graphs for the A375, SK-MEL-28, and RPMI-7951 in Fig 1. (ZIP)

S3 File Available cropped blots underlying the Figs 3, 4, and 5 results. (ZIP)
