# Supplementary material for: Expression of Concern: Fisetin Inhibits Human Melanoma Cell Invasion through Promotion of Mesenchymal to Epithelial Transition and by Targeting MAPK and NFκB Signaling Pathways
Source: PLoS One. 2024 Oct 28;19(10):e0313108. doi: 10.1371/journal.pone.0313108 (PMC11516002; doi:10.1371/journal.pone.0313108)
Supplement: S1 File — (PDF) [file pone.0313108.s001.pdf]

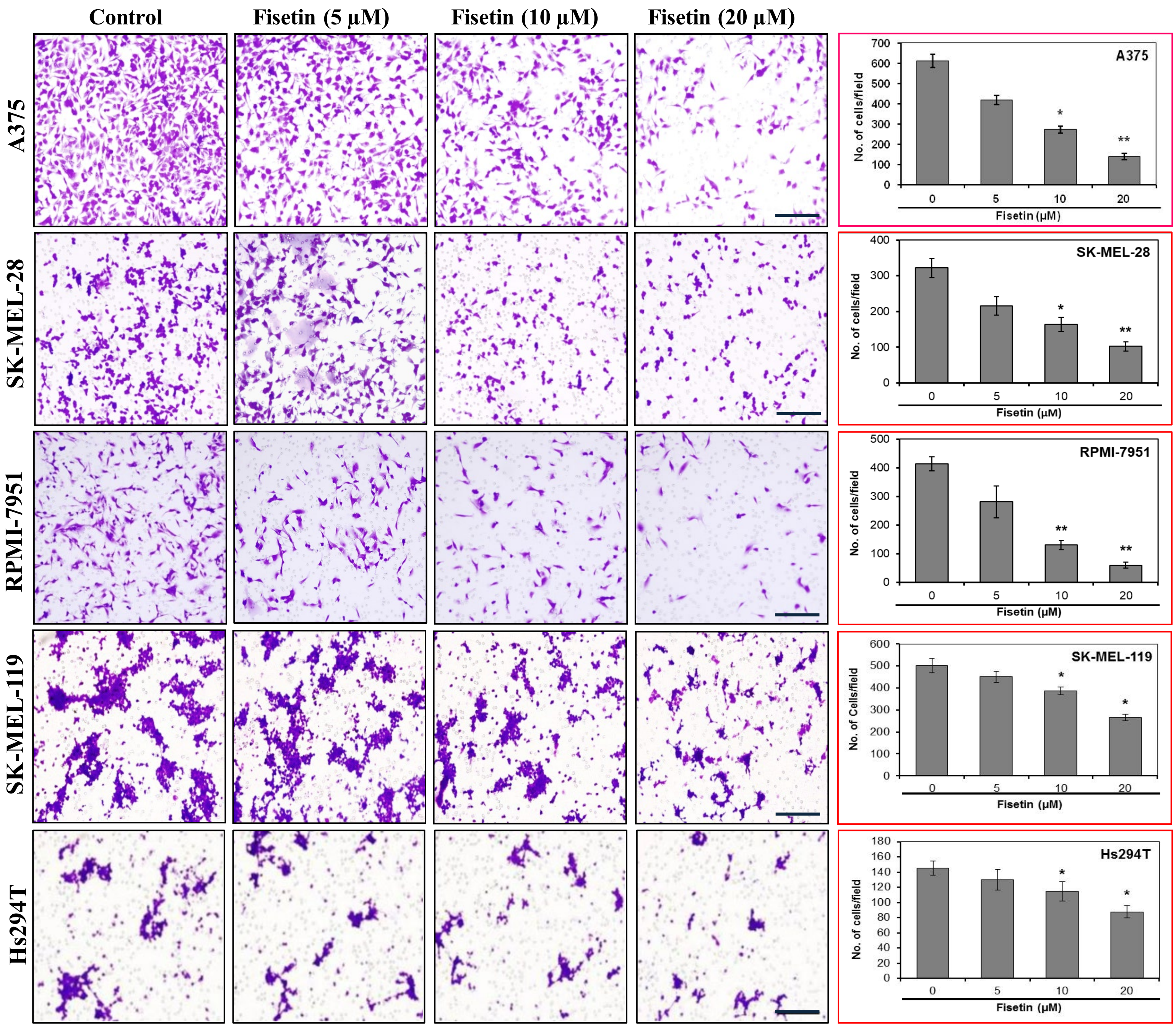
Control
Fisetin (5 µM)
Fisetin (10 µM)
Fisetin (20 µM)
A375
SK-MEL-28
RPMI-7951
SK-MEL-119
Hs294T
No. of cells/field
No. of Cells/field
Fisetin (µM)
*
**

Figure 1. Effect of fisetin treatment on invasion of melanoma cells. The invasive capacity of BRAF mutated (A375, SK-MEL-28 and RPMI-7951), NRAS mutated (SK-MEL-119) and BRAF-NRAS wild type (Hs294T) melanoma cells was determined *in vitro* using Boyden chamber assay. Melanoma cells (3×10^4 cells/200 µl serum-reduced medium) were placed in the upper chamber of Boyden chamber containing 0, 5, 10 and 20 µM of fisetin. The lower chamber contained 110 µl of medium supplemented with 10% FBS. After 24 hours of incubation, the invaded cells on the lower surface of the membranes were fixed with chilled methanol and stained with crystal violet. A representative picture from three independent experiments is shown. The invaded cells were counted in at least four to five randomly selected microscopic fields on the membrane and the results are summarized and expressed as the mean number of invaded cells ± SEM per microscopic field. Significant difference *versus* control group, *$P<0.05$, **$P<0.01$. Bar = 100 µm
