# Supplementary figures and images for: Expression of Concern: Fisetin Inhibits Human Melanoma Cell Invasion through Promotion of Mesenchymal to Epithelial Transition and by Targeting MAPK and NFκB Signaling Pathways
Source: PLoS One. 2024 Oct 28;19(10):e0313108. doi: 10.1371/journal.pone.0313108 (PMC11516002; doi:10.1371/journal.pone.0313108)

**A375 Cells**

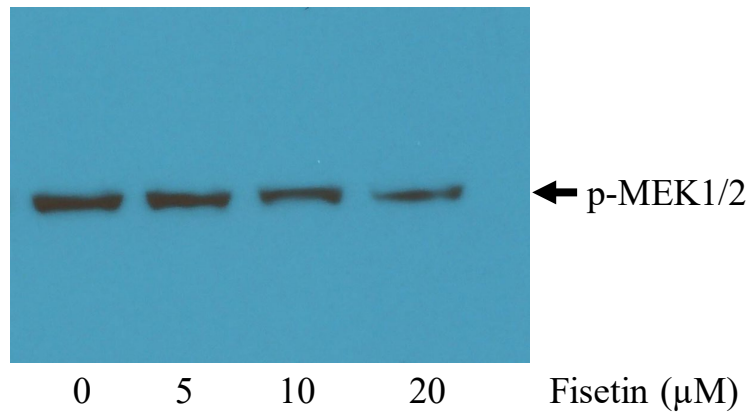

**RPMI-7951 Cells**

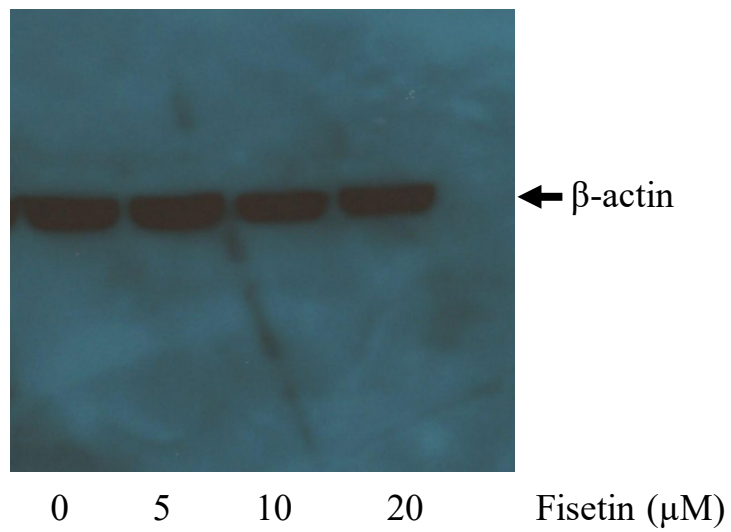

**Figure 3A**

Supplement: S3 File — (ZIP) [file pone.0313108.s003.zip › S3 File - Available blots underlying Figs 3, 4, and 5/Appendix-1.pdf]

### A375 Cells

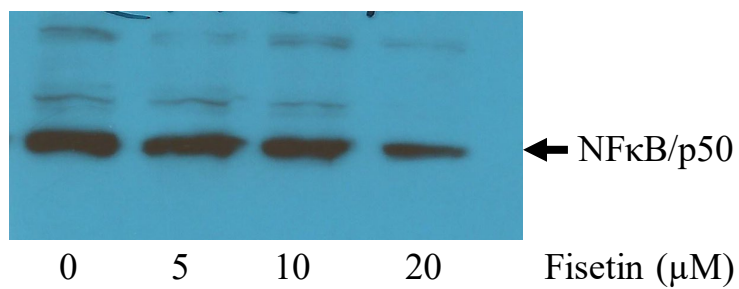

### RPMI-7951 Cells

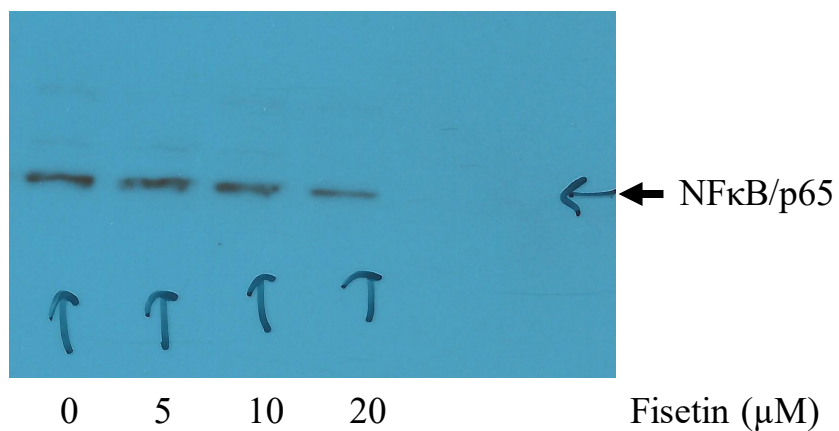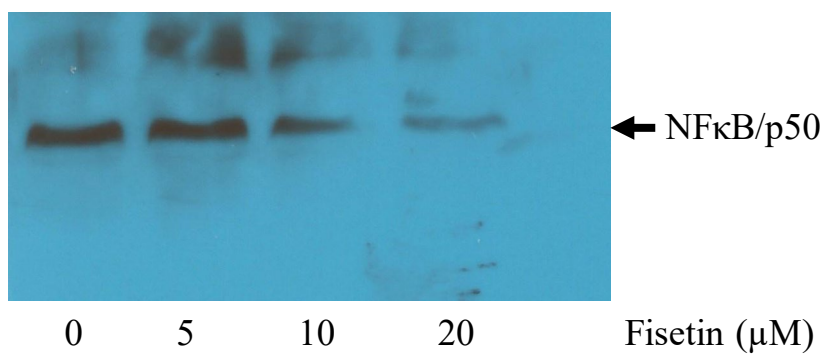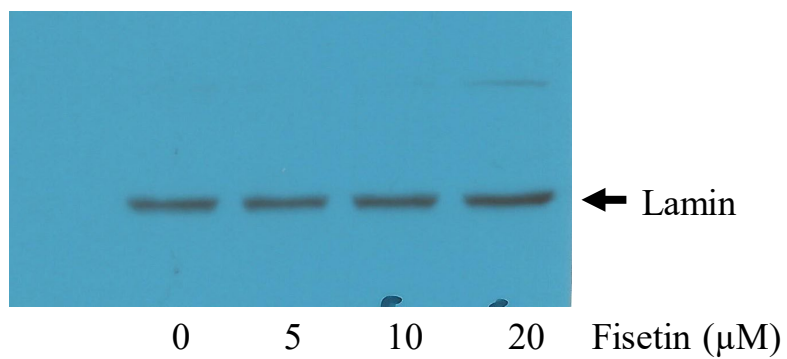

**Figure 4A**

**A375 Cells**

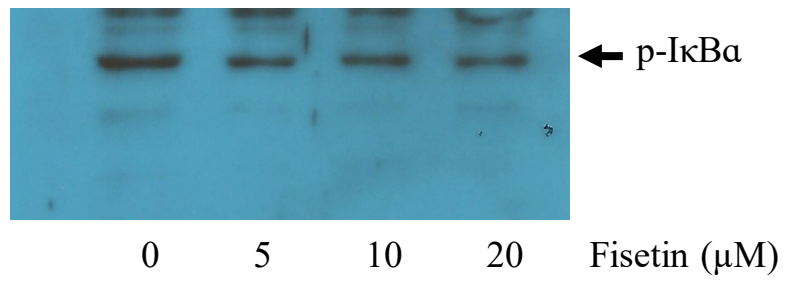

**Figure 4C**

Supplement: S3 File — (ZIP) [file pone.0313108.s003.zip › S3 File - Available blots underlying Figs 3, 4, and 5/Appendix-2.pdf]

**RPMI-7951 Cells**

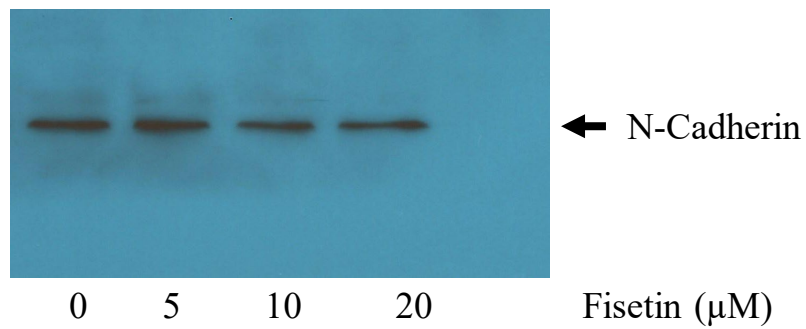

**A375 Cells**

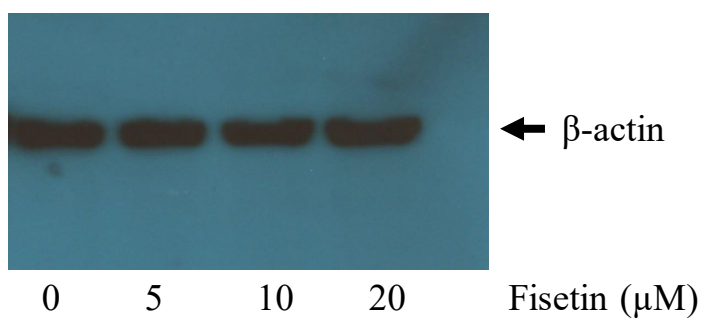

**Figure 5A**

Supplement: S3 File — (ZIP) [file pone.0313108.s003.zip › S3 File - Available blots underlying Figs 3, 4, and 5/Appendix-3.pdf]
